# Development of Single‐Molecule Enzyme Activity Assay for Serine Hydrolases Using Activity‐Based Protein Labeling Probes

**DOI:** 10.1002/smtd.202501643

**Published:** 2025-09-29

**Authors:** Seiya Ishii, Mayano Minoda, Tadahaya Mizuno, Takumi Iwasaka, Hiroyuki Kusuhara, Kazufumi Honda, Yasuteru Urano, Toru Komatsu

**Affiliations:** ^1^ Graduate School of Pharmaceutical Sciences The University of Tokyo Tokyo 113‐0033 Japan; ^2^ Institute for Advanced Medical Science, Graduate School of Medicine Nippon Medical School Tokyo 113‐8602 Japan; ^3^ Graduate School of Medicine Nippon Medical School Tokyo 113‐8602 Japan; ^4^ Graduate School of Medicine The University of Tokyo Tokyo 113‐8654 Japan

**Keywords:** chemical biology, enzymes, enzymomics, single‐molecule analysis

## Abstract

Single‐molecule enzyme activity assays have proven their potential in elucidating aberrant protein function at the proteoform level. However, the limited number of targetable enzymes is the major drawback of such assays. Here, the development of single‐molecule enzyme activity assays utilizes activity‐based probes that label active enzymes in an enzyme superfamily‐wide manner. A proof‐of‐principle using fluorophosphonate‐based probes is conducted to detect the active form of serine hydrolases such as PSA and granzyme B at the single‐molecule level in complex biological systems such as blood. The assay suggests that active granzyme B in blood may serve as an indicator of liver damage associated with immune cell activation

## Graphical abstract

This study introduces a single‐molecule strategy using activity‐based probes (ABPs) to sensitively detect accessible enzymes in complex biological systems. The approach successfully visualizes the active forms of PSA and granzyme B, demonstrating its utility for probing protease activity states with high selectivity and sensitivity at the molecular level.

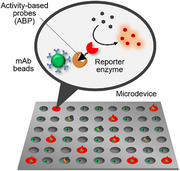

## 1 Introduction

**Figure 1:**
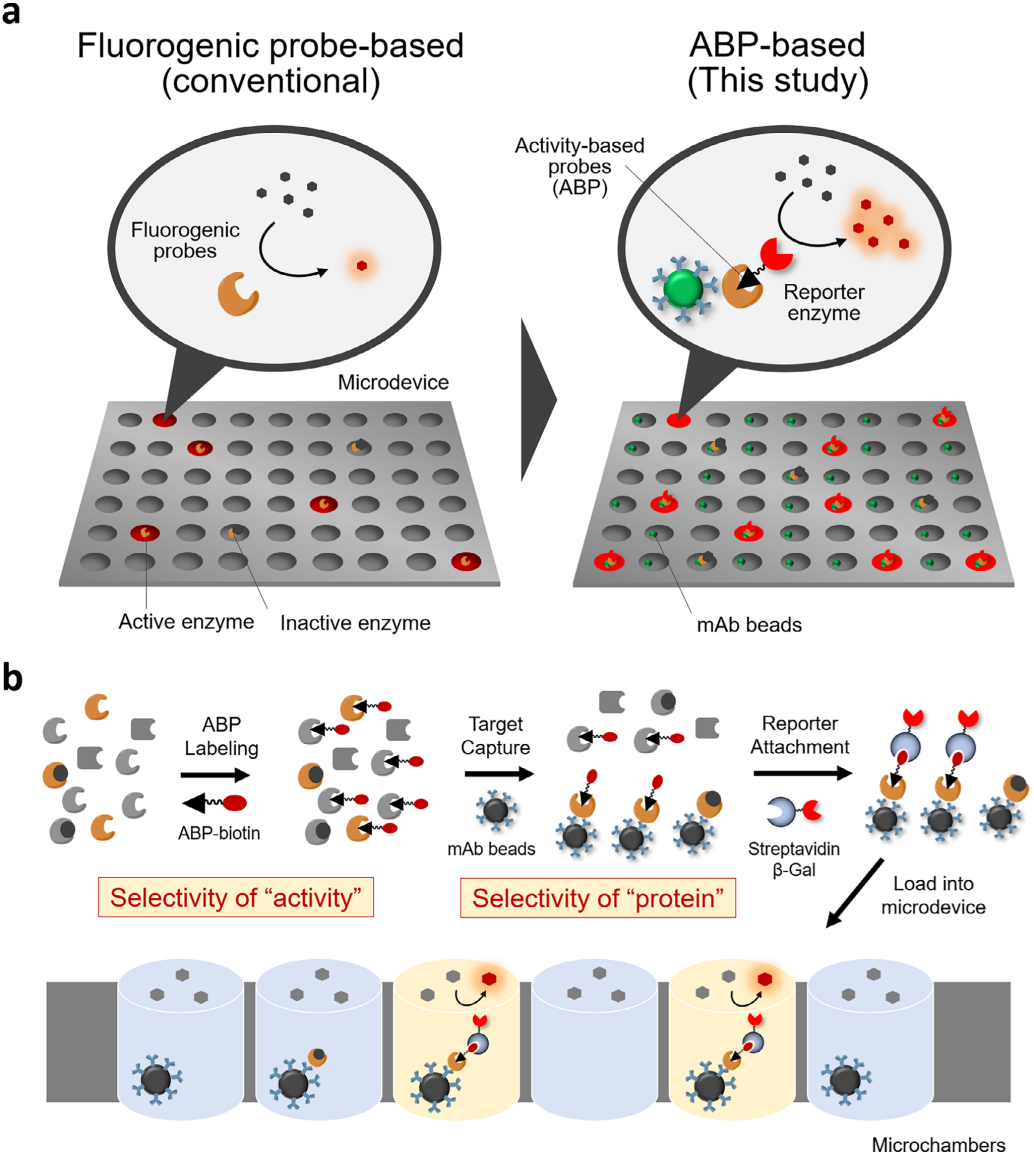
Schematic view of ABP‐based detection of enzymes with an accessible active site. a) Design of assay and comparison with the conventional fluorogenic probe‐based single‐molecule enzyme assays. b) Strategy to realize the selective detection of target enzymes with the accessible active site.

In living systems, there are thousands of enzymes involved in maintaining homeostasis, and their alterations are often tightly connected to the onset of diseases.^[^ ^1^, ^2^, ^3^, ^4^ ^]^ Enzyme activities are controlled at the transcriptional, translational, and post‐translational levels, and analysis of their functional alterations is useful for detecting and understanding diseases.^[^ ^3^, ^4^ ^]^ Application of single‐molecule analysis to detect disease‐related biomolecules has proven useful in disease diagnosis because of its high sensitivity.^[^ ^5^, ^6^, ^7^, ^8^, ^9^ ^]^ Extending this approach to study single‐molecule enzyme activities can provide a powerful system for understanding disease‐related alterations in protein functional states at the proteoform level.^[^ ^10^, ^11^, ^12^, ^13^, ^14^, ^15^ ^]^ The present single‐molecule enzyme assay employs fluorogenic substrate probes that are metabolized by enzymes to become fluorescent. However, the class of enzymes to which fluorogenic substrates produce sufficient signals remains limited,^[^ ^10^ ^]^ and the requirement for individual development of specific substrates is the bottleneck to broad assay deployment. In this study, we propose a general methodology that utilizes activity‐based protein labeling probes (ABPs) to detect single‐molecule enzymes with the active site accessible by ABPs (**Figure** **1**a).

ABPs are molecules that covalently label the active site of enzymes, enabling discrimination between active and inactive enzymes. Activity‐based protein profiling (ABPP) utilizes these characteristics for global analysis of enzyme activities.^[^ ^16^, ^17^ ^]^ We considered that exploiting these characteristics would allow single‐molecule assays to discriminate proteins depending on their activation state. The conventional single‐molecule enzyme activity assay is performed by capturing the single‐molecule target enzyme in a microfabricated chamber device, and the metabolism of the fluorogenic substrate generates a fluorescent signal in the chamber. The detection relies on enzymatic amplification of the signal, such that covalently attaching a reporter enzyme to the ABP can realize its readout. Since ABPs globally label various enzymes in the same superfamily, we combined the ABP with antibody‐based capture of the target enzyme for selective detection of the protein of interest (Figure 1b). The overall system is the extension of the digital ELISA platform, but the secondary antibody in digital ELISA was substituted by ABPs. It has two technical advantages: i) We can selectively detect enzymes with accessible active sites, rather than detecting the whole protein without discriminating its activity states in digital ELISA, ii) We do not need to prepare two antibody pairs with independent binding sites, which sometimes is a hard task in designing digital ELISA.

## 2 Development of ABP‐Based Single‐Molecule Assay for Prostate‐Specific Antigen (PSA)

**Figure 2:**
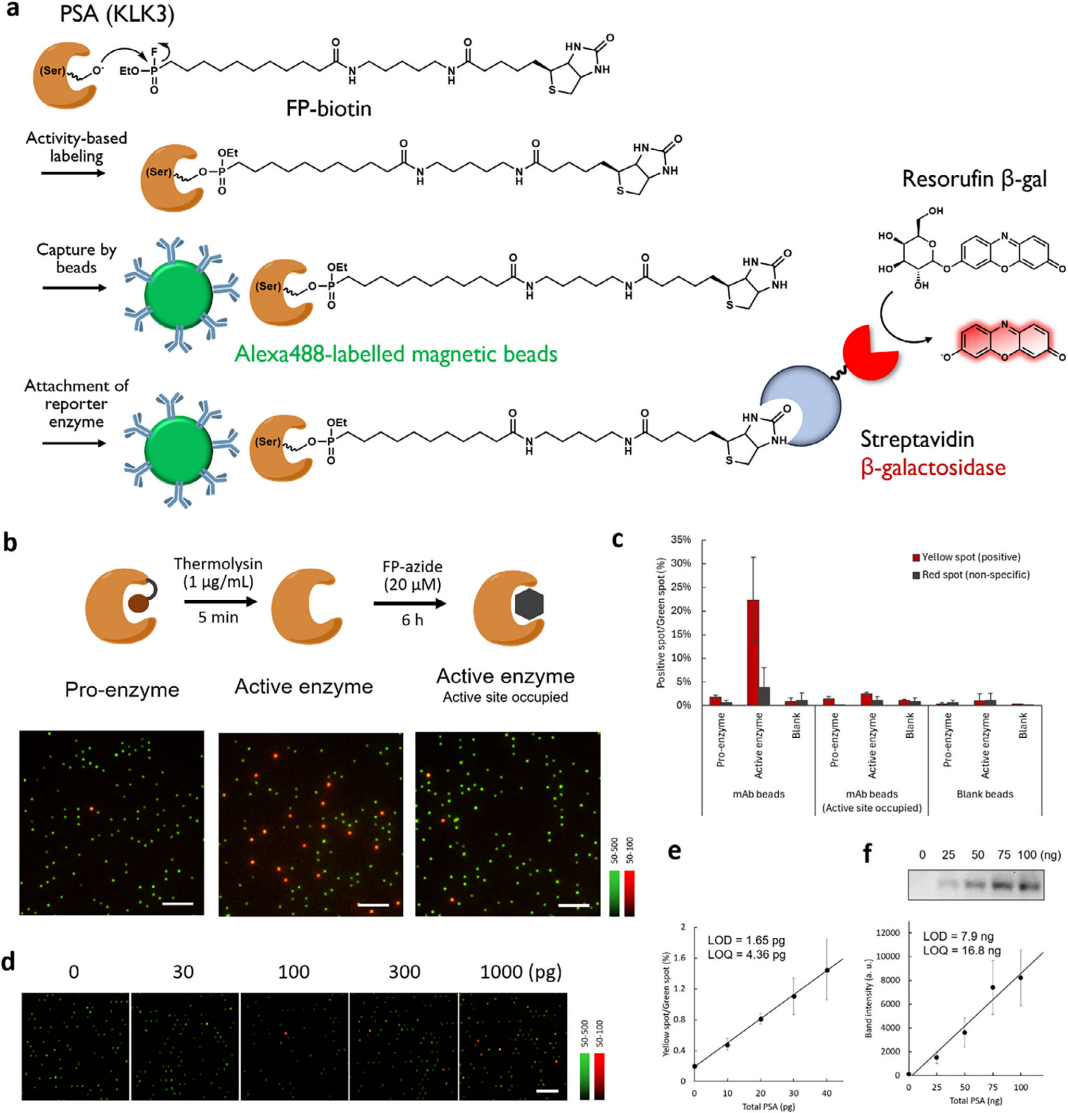
Construction of a single‐molecule enzyme activity‐based protein profiling assay using PSA. a) PSA labeling scheme for activity‐based probes, antibody capture, and streptavidin β‐Gal attachment for activity readout. b) Detection of single‐molecule active PSA in the pro‐enzyme form, active form, and inhibitor‐treated active‐form samples. Scale bar = 30 µm. c) Quantification of the result of Figure 2b. “Yellow spot” indicates the number of microchambers with green fluorescence (beads) and red fluorescence (β‐galactosidase) was detected. “Red spot” indicates the number of microchambers in which only red fluorescence was detected, so it was generated as a non‐specific binding of the conjugates. Error bars represent S. D. (*n* = 3). d) Fluorescence images of the microdevice loaded with various amounts of activated PSA. The labeling and loading conditions were the same as those in Figure 2b. Scale bar = 30 µm. e) Correlation between the proportion of yellow spots and activated PSA in Figure 2d. The amount of PSA refers to pro‐PSA used for the assay. Error bars represent S. D. (*n* = 3). The limit of detection (LOD) was calculated as the value corresponding to blank + 3σ. The limit of quantification (LOQ) was calculated as the value corresponding to blank + 10σ. f) LOD and LOQ were calculated in ABP labeling and chemiluminescence‐based detection by western blotting. Error bars represent S. D. (n = 3). The same protein samples were used in Figure 2d,e. The amount of PSA refers to pro‐PSA used for activation.

We initiated the proof‐of‐concept study of ABP‐based single‐molecule protein analysis using prostate‐specific antigen (PSA) as a biomarker of prostate cancer.^[^ ^18^ ^]^ Currently, antibody‐based detection is commonly used to measure PSA in blood as a biomarker of prostate cancer, and single‐molecule assays such as digital ELISA have also been applied; however, these methods detect the total amount of PSA molecules regardless of their active site accessibility. PSA (kallikrein 3) is a chymotrypsin‐like endopeptidase, and its activity contributes to various physiological processes such as reproduction, regulation of blood pressure, and inflammation.^[^ ^19^ ^]^ The enzyme is generated as a preproenzyme and must be correctly processed by cell surface proteases to become proteolytically active.^[^ ^20^ ^]^ Therefore, discriminately analyzing PSA by its activity states can offer useful insight for disease diagnosis, but the conventional single‐molecule enzyme assay using a specifically designed fluorogenic probe failed to report the activity (Figure S1, Supporting Information), presumably due to the insufficient reactivity of the probe. Since the active site of serine hydrolases can be selectively labeled by fluorophosphonate (FP),^[^ ^16^, ^21^ ^]^ we have used FP‐based ABPs to construct a system to selectively detect the active form of PSA (**Figure** **2**a). After the activity‐based labeling of PSA with FP‐biotin, it was captured by an anti‐PSA antibody bound to fluorescent magnetic beads (green fluorescence; labeled by Alexa Fluor 488), and biotin was labeled with streptavidin β‐Gal (SA β‐Gal). The complex was loaded into a microdevice, and the number of chambers containing β‐galactosidase activity was read out by the fluorescence of resorufin β‐gal (red fluorescence). Loading of the complex was automated using the digital ELISA instrument siMoA,^[^ ^22^ ^]^ and detection of the fluorescent complexes was performed using a standard epifluorescence microscope. The assay selectively detected PSA activated by thermolysin (active form, Figure S2, Supporting Information), as the increase of red fluorescent spots reflecting β‐galactosidase activity. The spot number was significantly lower for the preproenzymes (pro‐form) or enzymes treated with inhibitors (Figure 2b,c), confirming that the assay results reflected the number of enzymes with accessible active sites rather than total protein.

**Figure 3:**
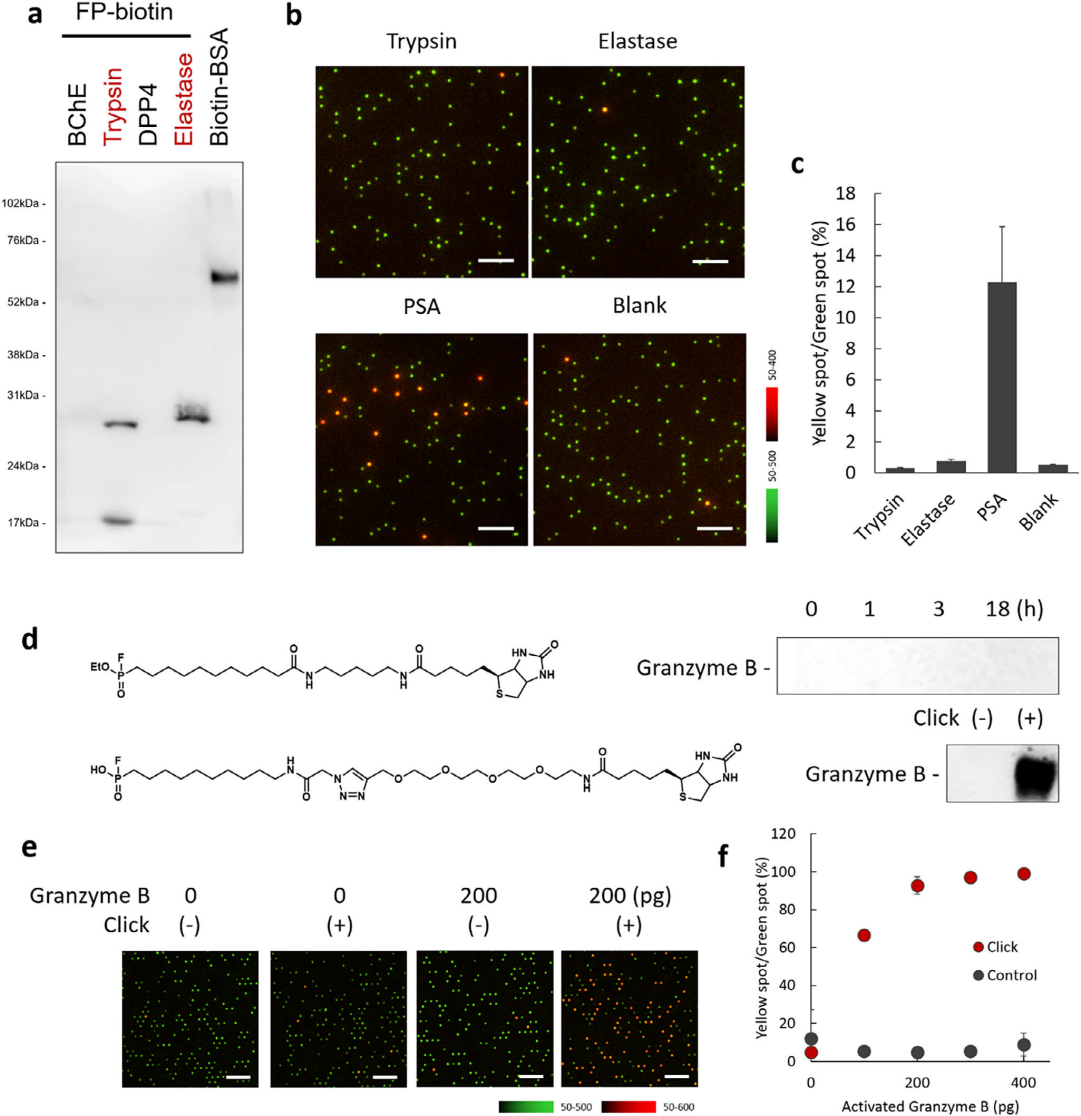
Extension of the target of ABP‐based single‐molecule analysis to other serine hydrolases. a) Western blot analysis of serine hydrolases labeled with FP‐biotin. Biotin‐modified BSA was used as a positive control. Enzymes with the positive signal observed with FP‐biotin were red lettered. b) Representative fluorescence overlay images of the microdevice containing recombinant enzymes (trypsin, elastase, activated PSA) or blank. The protocol was the same as that of Figure 2b. Scale bar = 30 µm. c) Quantification of the result of Figure 3b. Error bars represent S. D. (*n* = 3). d) FP‐biotin probes with different linker lengths for granzyme B labeling. Western blot analysis of activated granzyme B. For labeling with FP‐biotin (top), the labeling/detection condition was the same as that in Figure 3a. For labeling with FP‐azide (bottom), granzyme B was labeled with FP‐azide, and the sample was reacted with alkyne‐PEG4‐biotin for click reaction. e) Detection of recombinant granzyme B using FP‐azide. The labeling was performed under the same conditions as in Figure 3d. Scale bar = 30 µm. f) Quantification of the result in Figure 3e. The amount of activated granzyme B refers to the granzyme B used for the assay. Error bars represent S. D. (*n* = 3).

The advantage of the single‐molecule assay is its high detection sensitivity compared with conventional protein detection platforms performed under bulk conditions. When the same protein sample was detected in a single‐molecule assay platform and SDS‐PAGE/chemiluminescence detection after capturing FP‐biotin by streptavidin (SAv, attached to β‐galactosidase in the single‐molecule assay and attached to horseradish peroxidase (HRP) in chemiluminescence detection), the single‐molecule assay had a limit of detection (LOD) that is >1,000‐times lower than that of SDS‐PAGE/chemiluminescence detection (Figure 2d–f). The increased sensitivity is attributed to the combination of the high sensitivity of the single‐molecule analysis itself and antibody enrichment of the target (Figure 1). It should be noted that, in this assay, PSA is detected after activation from its proenzyme form, and since the activation efficiency is undefined, comparison with the LOD of commercial total PSA assays^[^ ^23^ ^]^ (Figure S3, Supporting Information) is not straightforward. Nonetheless, we consider that the detection sensitivity for active PSA can be comparable to that of the digital ELISA assay for total PSA, since the enrichment by antibody beads operates under the same principle. The assay showed sufficient selectivity toward other serine hydrolases, such as trypsin and elastase, based on the selectivity of the capture antibody (Figure 1b); proteases that were labeled by FP‐biotin, such as trypsin and elastase, were not captured by the antibody‐modified beads and did not generate red fluorescent spots (**Figure** **3**a–c).

## 3 Development of ABP‐Based Single‐Molecule Assay for Granzyme B

Due to the generality of the assay scheme, it can target various serine hydrolases by changing the capture antibodies. We next developed another single‐molecule activity assay targeting granzyme B, which is generated by natural killer (NK) cells and CD8^+^ T cells.^[^ ^24^ ^]^ Because of its weak reactivity and low abundance in blood, granzyme B activity has not been reported in blood samples. We designed the fluorogenic probe that reacts with granzyme B, but its single‐molecule activity was not monitorable in the microdevice‐based assay (Figure S1, Supporting Information). As granzyme B is also a serine hydrolase, we predicted that active granzyme B could be detected by FP‐based labeling. The detection of granzyme B required the optimization of the ABP linker length; ABP with a short linker (FP‐biotin) failed to form a complex with granzyme B and SAv, so we designed to extend the linker between FP and biotin by labeling granzyme B using FP‐azide and attaching another linker biotin‐PEG4‐alkyne using click chemistry. In this linker extension strategy, SAv can form a complex with granzyme B labeled by ABPs, so the signal of SAv‐HRP‐based chemiluminescence was observed in SDS‐PAGE/chemiluminescence assay (Figure 3d). We consider that this is due to structural constraints, such as the depth of the granzyme B binding pocket and steric hindrance around SAv, preventing the two proteins from coming close enough when using ABPs with short linkers.^[^ ^25^ ^]^ By employing the click‐chemistry‐based linker extension strategy, we were able to detect active granzyme B at the single‐molecule level (Figure 3e,f).

## 4 Detection of PSA and Granzyme B in Blood Samples of Mice with Liver Damage

**Figure 4:**
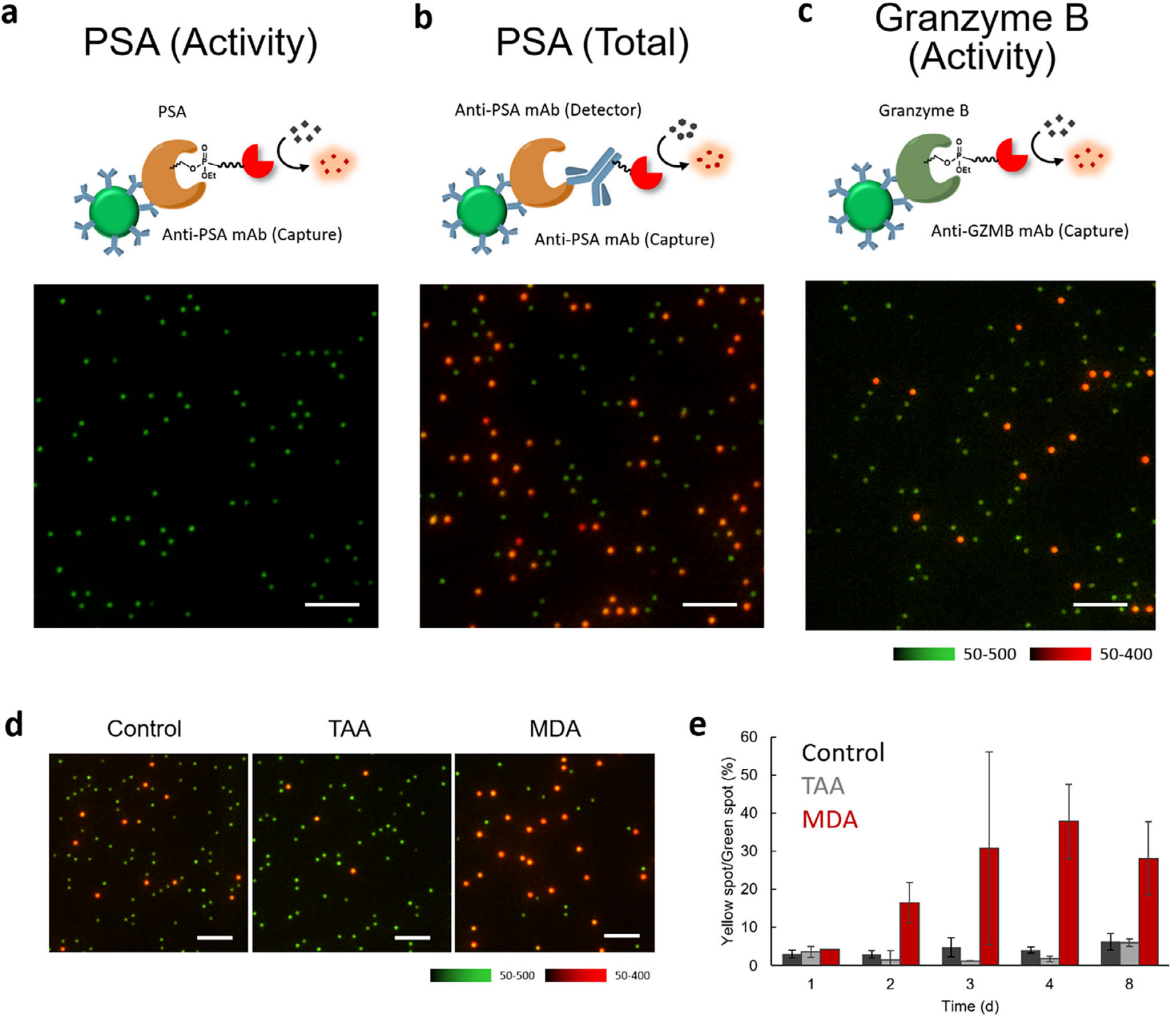
Analysis of serine hydrolase activities in blood using sABPP. a) Detection of active PSA in plasma samples from healthy human subjects (male, 1/300 dilution) using the same condition as in Figure 2b. Scale bar = 30 µm. b) Detection of total PSA in plasma samples from healthy human subjects (male, 1/300 dilution) using a digital ELISA platform. Biotin‐labeled secondary antibody was used instead of labeling with FP‐biotin. Scale bar = 30 µm. c) Detection of active granzyme B in plasma samples from healthy human subjects (male, 1/300 dilution) using the same condition as in Figure 3e. Scale bar = 30 µm. d) Detection of active granzyme B in plasma samples of mice treated with/without thioacetamide (TAA) as an acute liver injury model or 4,4’‐methylene dianiline (MDA) as a cholestatic liver dysfunction model. Plasma samples collected at 4 days of treatment were used. The plasma samples were diluted 1/300 for the assay. Scale bar = 30 µm. e) Time‐course changes of active granzyme B in liver damage models. Error bars represent S. D. (*n* = 3).

We then determined whether active PSA and granzyme B are present in blood, with the aim of developing them as activity‐based biomarkers. Active PSA was not observed in plasma samples from healthy human subjects (**Figure** **4**a). When total PSA in the same blood sample was measured using a detection antibody conjugated with the reporter enzyme (Figure 4b),^[^ ^23^ ^]^ red fluorescent spots reflecting the secondary antibody were observed. The result indicates that inactive PSA was the dominant form in blood due to the lack of activation by processing enzymes or inhibition by anti‐proteases (e.g., serpins).^[^ ^20^ ^]^ Active PSA was spiked into plasma samples before labeling, and the number of red fluorescent spots decreased, supporting the idea that anti‐proteases for PSA are dominant in blood samples of healthy human subjects (Figure S4, Supporting Information). In contrast, active granzyme B was detected in plasma samples of healthy human subjects (Figure 4c). We determined whether the number of granzyme B in blood samples is altered under different health conditions, with the aim of developing an activity‐based disease biomarker. Since granzyme B is generated exclusively in natural killer (NK) cells and CD8^+^ T cells, we expected that its activity could be altered by specific immune responses. To investigate this idea, we prepared two mouse models of liver damage by treating subjects with thioacetamide (TAA)^[^ ^12^, ^26^ ^]^ or 4,4′‐methylenedianiline (MDA),^[^ ^27^ ^]^ which are known to cause different forms of immune responses in the liver. The TAA‐model leads to acute liver damage and gradual liver fibrosis,^[^ ^26^ ^]^ while the MDA‐model leads to increased monocyte‐derived macrophages and enhanced fibrinolysis in liver.^[^ ^27^, ^28^ ^]^ While the mechanisms are different, the present biomarker AST/ALT, which is elevated during liver damage as tissue leakage products, cannot discriminate between these two liver damage models (Figure S5, Supporting Information).^[^ ^28^ ^]^ Interestingly, elevated levels of red spots were observed exclusively in blood samples of MDA‐treated mice (Figure 4d,e, S6, Supporting Information). We interpreted this as a reflection of an increase in granzyme B caused by an increased CD8^+^ T cells in MDA‐treated mice.^[^ ^28^ ^]^ The overall results indicate that activity‐based biomarker detection by single‐molecule enzyme activity assay can selectively detect specific forms of liver damage. Since granzyme B activity is important for tumor immunity,^[^ ^29^ ^]^ we contend that alterations in granzyme B activity can serve as a meaningful blood biomarker to evaluate changes in the immune status of tumor tissues, thereby contributing to early tumor detection and better classification of patients that respond to immune checkpoint inhibitors.

## 5 Conclusion

In summary, we report a methodology to utilize ABPs to selectively detect single‐molecule enzymes with an accessible active site. A wide variety of ABPs are currently being developed for enzyme families such as serine hydrolases, cysteine hydrolases, glycosidases, phosphatases, and kinases,^[^ ^16^, ^17^ ^]^ and their use in mapping the reactive landscape of proteins has also been proposed.^[^ ^30^, ^31^, ^32^ ^]^ In combination with these advances in ABP development, the assay scheme presented here has the potential to expand the range of protein activities accessible to single‐molecule analysis. In the future, multiplexed detection of related enzymatic activities and reactivities at the single‐molecule level will likely become feasible, increasing the likelihood of identifying disease‐associated proteoforms. Overall, the system provides a sensitive and versatile framework for detecting functional proteins relevant to disease diagnosis and the elucidation of biological mechanisms.

## 6 Experimental Section

### Plasma Samples from Healthy Human Subjects and Ethics Statement

Plasma samples from healthy human subjects were collected from Kagoshima Prefectural Comprehensive Health Center with the Program for Promotion of Fundamental Studies in Health Sciences conducted by the National Institute of Biomedical Innovation of Japan, Health and Labour Sciences Research Grants from the Ministry of Health, Labour and Welfare of Japan, and P‐CREATE of the Japan Agency for Medical Research and Development (AMED). Ethical approval from the central ethics committees of Nippon Medical School (M‐2021‐002) and the ethical committee of Nippon Medical School (A‐2020‐032 and A‐2020‐044), and informed written consent of all participants were obtained.

### Plasma Samples from Mice and Ethics Statement

Ethical approval for the study using animals was obtained from the Animal Care and Use Committee of The University of Tokyo (P4‐21, P31‐9). Six‐week‐old male C57BL/6JJcl mice were purchased from CLEA Japan (Tokyo, Japan) and acclimatized for five days. The mice were exposed to thioacetamide (TAA, TCI, T0817, 300 mg L^−1^) or 4,4'‐methylenedianiline (TCI M0220, 750 mg L^−1^) dissolved in drinking water to induce liver damage, whereas the controls received tap water. After four days of treatment, mice were euthanized, and the blood was collected through the inferior vena cava into a 1.5 mL tube containing 1.5 µL heparin (Yoshindo). The collected blood sample was centrifuged (1,700 g, 4 °C for 15 min) for plasma separation. Plasma alanine aminotransferase (ALT) and aspartate aminotransferase (AST) were measured using a DRI‐CHEM NX500sV (Fujifilm).

### Activation of PSA

Recombinant human pro‐PSA (50 µg mL^−1^; R&D Biosystems 1344‐SE) was mixed with recombinant thermolysin (1 µg mL^−1^; R&D Biosystems 3097‐Zn) in HEPES‐Na buffer (100 mm, pH 7.4) containing NaCl (150 mm) and CHAPS (0.1%), and the mixture was incubated at 37 °C for 5 min. 1,10‐Phenanthroline was added with a final concentration of 20 mm. The activated enzyme solution was either used immediately for the assay or aliquoted for single use, fresh frozen in liquid N_2,_ and maintained at −80 °C.

### Activation of Granzyme B

Recombinant human granzyme B (20 µg mL^−1^; R&D Biosystems 2906‐SE) was mixed with recombinant mouse cathepsin C (5 mg mL^−1^; R&D Biosystems 2336‐CY‐) in HEPES‐Na buffer (10 mm, pH 5.5), and the mixture was incubated at 37 °C for 4 h. The mixture was diluted 1/10 with HEPES‐Na buffer (100 mm, pH 7.4) containing NaCl (150 mm) and CHAPS (0.1%). The activated enzyme solution was either used immediately for the assay or aliquoted for single use, fresh frozen in liquid N_2_, and maintained at −80 °C.

### Preparation of Antibody‐Conjugated Beads

The magnetic beads conjugated with antibodies were prepared using reagents and protocols of the Simoa Homebrew assay starter kit (Quanterix). For fluorescent magnetic beads, the Simoa 488‐dyed Singleplex Bead (Quanterix) was used. For PSA, the unconjugated capture antibody of the anti‐PSA antibody pair for ELISA (abcam; ab256313) was used. For granzyme B, the unconjugated detector antibody of the anti‐granzyme B antibody pair for ELISA (abcam; ab245038) was used. First, the buffer of the antibody solution was exchanged to Bedas conjugation buffer (Quanterix) by three rounds of buffer exchange (loading of the 500 µL solution in an Amicon ultra (50K) and centrifuging at 14,000 rcf × 5 min, 25 °C). The concentration of the antibody was measured using a Nanodrop One (Thermo Fisher Scientific), and the solution was diluted to 0.2 mg mL^−1^ with Antibody conjugation buffer. 4.2 × 10^8^ magnetic beads were washed three times with 300 µL Bead wash buffer (Quanterix) by capturing beads on a magnetic stand (Takara Bio). The beads were dissolved in 291 µL Bead conjugation buffer. A 0.1 mg mL^−1^ solution of 1‐(3‐Dimethylaminopropyl)‐3‐ethylcarbodiimide (EDC) was freshly prepared, and 9 µL was added while vortexing. After shaking at 4 °C for 30 min, the supernatant was removed, magnetic beads were quickly washed with Bead conjugation buffer, and 300 µL antibody solution (0.2 mg mL^−1^) was added while vortexing. The mixture was incubated at 4 °C for 2 h. The beads were washed with 300 µL Bead wash buffer twice, and shaken in 300 µL Bead blocking buffer (Quanterix) at 25 °C for 45 min. The beads were washed with 300 µL Bead wash buffer and stored in 300 µL Bead diluent buffer (Quanterix).

### Fluorophosphonate (FP)‐Biotin‐Based Enzyme Labeling and Analysis

The sample was diluted in HEPES‐Na buffer (100 mm, pH 7.4) containing NaCl (150 mm) and CHAPS (0.1%), FP‐biotin (Santa Cruz sc‐215056) was added with a final concentration of 20 µm, and the reaction was incubated at 25 °C for 14 h. Then, the sample was diluted to 1/100 in HEPES‐Na buffer (100 mm, pH 7.4) containing NaCl (150 mm) and CHAPS (0.1%), and a 100 µL volume was mixed with 25 µL of antibody‐conjugated beads (2 × 10^7^ beads mL^−1^; diluted with Bead diluent buffer (Quanterix)) in a 96‐well plate (Thermo Fisher Scientific 249944) and shaken (800 rpm, 30 °C) for 30 min using a Microplate shaker (Quanterix). The plate was then washed with a microplate washer (BIOTEK 405 TS). To the remaining beads, 100 µL of streptavidin β‐Gal (100 pm, diluted with SBG Diluent buffer; Quanterix) was added and incubated at 30 °C for 10 min. The plate was washed with the microplate washer (BIOTEK 405 TS). In SR‐X (Quanterix), the beads were mixed with a solution of resorufin β‐Gal (10 µm) in HEPES‐Na buffer (100 mm, pH 7.4) containing CaCl_2_ (1 mm), MgCl_2_ (1 mm), and Triton‐X‐100 (250 µm) and loaded into a Simoa disk (Quanterix) following the standard protocol. After incubation at 25 °C for 1 h, fluorescence images of the Simoa disk were acquired using an epifluorescence microscope.

### FP‐Azide‐Based Enzyme Labeling, Click Conjugation, and Analysis

FP‐azide (ActivX #88316) was added to a sample diluted in HEPES‐Na buffer (100 mm, pH 7.4) containing NaCl (150 mm) and CHAPS (0.1%) at a final concentration of 40 µm, and the reaction was incubated at 25 °C for 18 h. The sample was then diluted 1/10 in HEPES‐Na buffer (100 mm, pH 7.4) containing NaCl (150 mm) and CHAPS (0.1%), and alkyne‐PEG4‐biotin (10 µm), CuSO_4_ (1 mm), BTTP (3 mm), and sodium ascorbate (1 mm). In the control condition, alkyne‐PEG4‐biotin (10 µm) was added without other reagents, and the reaction was incubated at 25 °C for 2 h. The sample was then diluted 1/10 in HEPES‐Na buffer (100 mm, pH 7.4) containing NaCl (150 mm) and CHAPS (0.1%), and a 100 µL volume was mixed with 25 µL of antibody‐conjugated beads (2 × 10^7^ beads mL^−1^; diluted with Bead diluent buffer (Quanterix)) in a 96‐well plate (Thermo Fisher Scientific 249944) and shaken (800 rpm, 30 °C) for 30 min using the Microplate shaker (Quanterix). The plate was then washed with the microplate washer (BIOTEK 405 TS). To the remaining beads, 100 µL of streptavidin β‐Gal (100 pm, diluted in SBG Diluent buffer; Quanterix) was added, and incubated at 30 °C for 10 min. The plate was washed with the microplate washer (BIOTEK 405 TS). In SR‐X (Quanterix), the beads were mixed with resorufin β‐Gal (10 µm) in HEPES‐Na buffer (100 mm, pH 7.4) containing CaCl_2_ (1 mm), MgCl_2_ (1 mm), and Triton‐X‐100 (250 µm) and loaded into a Simoa disk (Quanterix) following the standard protocol. After incubation at 25 °C for 1 h, fluorescence images of the Simoa disk were acquired using an epifluorescence microscope.

### Digital ELISA

Digital ELISA to detect PSA was performed using the Simoa technology (Quanterix) following the manufacturer's protocol. The sample was diluted to 1/100 in HEPES‐Na buffer (100 mm, pH 7.4), and 100 µL was mixed with 25 µL of antibody‐conjugated beads (2 × 10^7^ beads mL^−1^; diluted with Bead diluent buffer (Quanterix)) in a 96‐well plate (Thermo Fisher Scientific 249944) and shaken (800 rpm, 30 °C) for 30 min using a Microplate shaker (Quanterix). The plate was then washed with a microplate washer (BIOTEK 405 TS). To the remaining beads, 100 µL of biotinylated secondary antibody (250 ng mL^−1^) was added and shaken (800 rpm, 30 °C) for 10 min using a Microplate shaker (Quanterix). The plate was washed with a microplate washer (BIOTEK 405 TS). To the remaining beads, 100 µL of streptavidin β‐Gal (100 pm, diluted in SBG Diluent buffer; Quanterix) was added, and incubated at 30 °C for 10 min. The plate was washed with a microplate washer (BIOTEK 405 TS). In SR‐X (Quanterix), the beads were mixed with resorufin β‐Gal (10 µm) in HEPES‐Na buffer (100 mm, pH 7.4) containing CaCl_2_ (1 mm), MgCl_2_ (1 mm), and Triton‐X‐100 (250 µm), and loaded into a Simoa disk (Quanterix) following the standard protocol. After incubation at 25 °C for 1 h, fluorescence images of the Simoa disk were acquired using an epifluorescence microscope.

### Enzyme Activity Assay using Microplate Reader

The enzyme activity assay was performed in HEPES‐Na buffer (100 mm, pH 7.4) containing CHAPS (0.1%). The fluorometric assay was performed using half‐area 384‐well plates (Greiner 784900) (20 µL reaction volume). The absorbance assay was performed using 96‐well clear plates (Thermo Fisher Scientific 167008). The signals were acquired with a plate reader, Envision 2103 Multilabel Reader (Perkin Elmer), with appropriate filter settings.

### FP‐Based Enzyme Labeling and Western Blotting

To the sample diluted in HEPES‐Na buffer (100 mm, pH 7.4), FP‐biotin was added with a final concentration of 20 µm, and the reaction was incubated at 25 °C for 14 h. The samples were diluted in HEPES‐Na buffer (100 mm, pH 7.4) and mixed with the same amount of Laemmli sample buffer containing β‐mercaptoethanol (2×), and denatured at 95 °C for 3 min. For SDS‐PAGE, an 8 µL sample (40 µg total protein) was loaded onto a Multigel mini 10/20 (17 wells, Cosmo Bio) and separated using a 30 mA gel^−1^ constant current condition for 1 h. ECL Rainbow marker (Cytiva) was used as the protein ladder. After electrophoresis, the gels were transferred to PVDF membranes using a semi‐dry blotting kit (Protein Transfer Kit for Semidry Electroblot, Cosmo Bio) following the standard protocol. Electrophoresis was performed using a 100 mA gel^−1^ constant current condition for 1 h. The membrane was washed three times with Tris‐buffered saline containing Tween‐20 (0.05%; TBS‐T), and incubated with Streptavidin‐HRP (Cell Signaling Technology #3999, 1/3000 in TBS‐T) for 1 h. The membrane was washed three times with TBS‐T. The HRP‐linked antibody was detected using a chemiluminescence reagent (Westar Supernova, Cyanagen) with a gel imager (ImageQuant LAS 4000 Mini, Cytiva).

### Statistical Analysis

For data requiring statistical analysis, the experiments were performed with indicated replicates (n), and the data are presented as mean ± S. D. (error bars). Statistical analyses were performed with Student's *t*‐test (two‐sided test, equal error variances) using Microsoft Excel.

### Epifluorescence Microscopy

Fluorescence images were acquired using a fluorescence microscope (Ti2, Nikon) equipped with a 20× dry objective lens (Plan Apo 20×), sCMOS camera (ORCA‐Fusion C14440, Hamamatsu Photonics), white LED illumination unit (X‐Cite Xylis, Opto Science), and a motorized stage. Images were acquired in tile scan mode with perfect focus. The excitation and emission filters used were FITC (mirror = 510 nm, Ex. = 460–500 nm, Em. = 510–560 nm) and mCherry (mirror = 600 nm, ex. = 550–590 nm, Em. = 608–683 nm).

### Image Processing

Images were processed using the GA3 module of NIS Elements software (Nikon). First, all fluorescence images were background‐corrected using a rolling ball correction (3 µm). Then, ROIs were chosen by bright spot detection using the FITC and mCherry filters (diameter = 3 µm), and irregular fluorescent spots derived from fluorescent debris or air bubbles were omitted by dilating the ROI and removing the overlapping ROIs using a size and shape filter. The number of spots was counted as the number of ROIs in each channel.

### Synthesis of Turnover‐Based Fluorogenic Probes for PSA and Granzyme B

The fluorescent probes used in this study were synthesized on the basis of the fluorophore scaffold previously reported.^[^ ^33^ ^]^ The probes were prepared by solid‐phase synthesis following a general conjugation strategy to attach the recognition moiety to the fluorophore. Detailed synthetic procedures and full characterization will be reported separately.

### Statistical Analysis

For experiments with replicates, data are presented as mean ± SD, and the number of replicates (n) is indicated in the figure legends. No statistical significance tests were performed in this study.

## Conflict of Interest

The authors declare no conflict of interest.

## Supporting information



Supporting Information

## Data Availability

The data that support the findings of this study are available from the corresponding author upon reasonable request.
