# Supplementary material for: Development of Single‐Molecule Enzyme Activity Assay for Serine Hydrolases Using Activity‐Based Protein Labeling Probes
Source: Small Methods. 2025 Sep 29;9(11):e01643. doi: 10.1002/smtd.202501643 (PMC12641359; doi:10.1002/smtd.202501643)
Supplement: Supplementary file 1 — Supporting Information [file SMTD-9-e01643-s001.docx]

Supporting Information

Development of single-molecule enzyme activity assay for serine hydrolases using activity-based protein labeling probes

Seiya Ishii^1^, Mayano Minoda^1^, Tadahaya Mizuno^1^, Takumi Iwasaka^1^, Hiroyuki Kusuhara^1^, Kazufumi Honda^2,3^, Yasuteru Urano^1,4*^, and Toru Komatsu^1*^

**Supporting Figures**


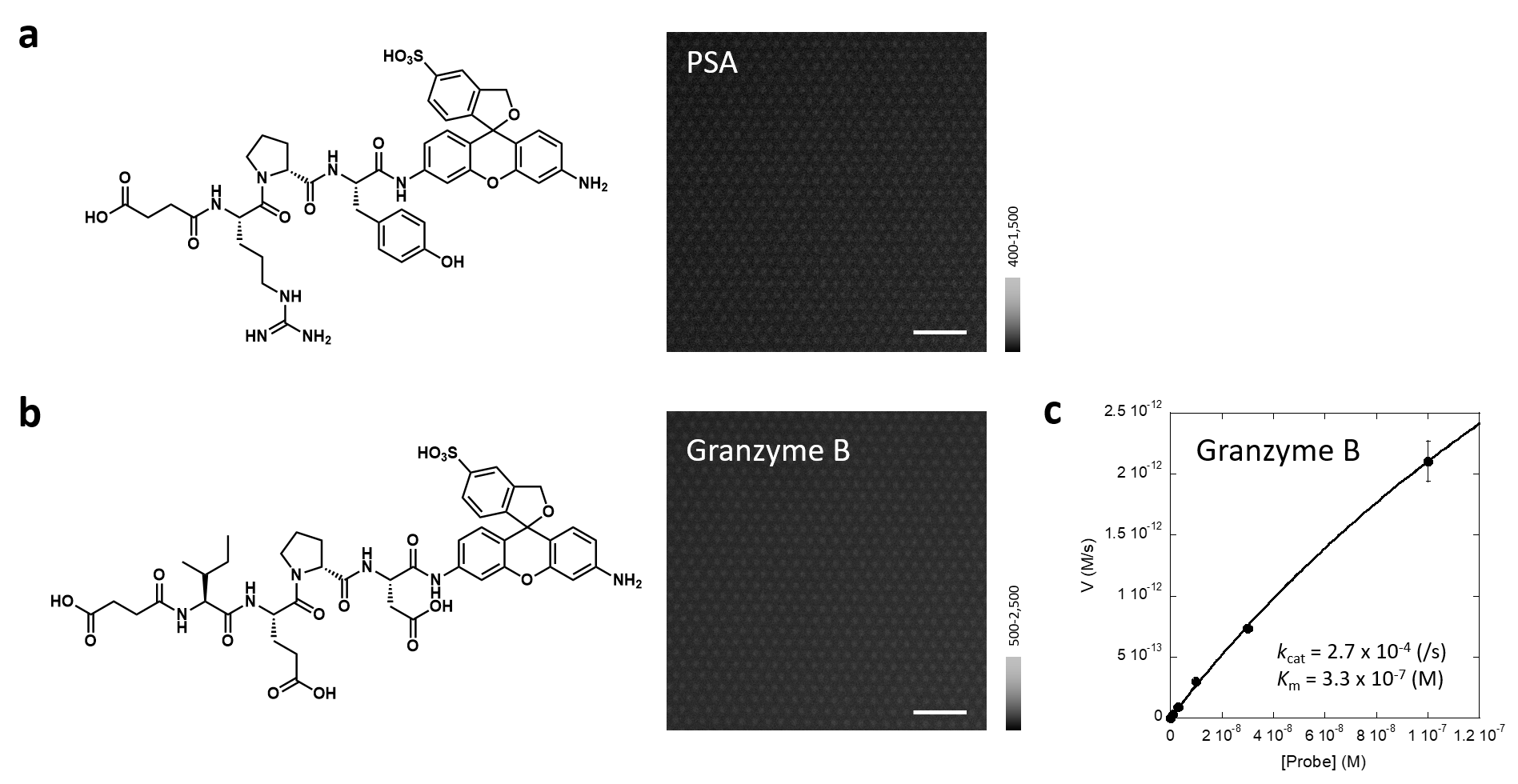


**Figure S1.** Detection of PSA and granzyme B using conventional fluorogenic probe-based single-molecule assays. The core scaffold of fluorogenic probes suitable for microdevice-based assay^33^ was modified with the peptide sequences known to target PSA (Arg-Pro-Tyr) and granzyme B (Ile-Glu-Pro-Asp). (a) Fluorescence images of microdevice after loading the Suc-Arg-Pro-Tyr-sHMRG (30 μM) and activated PSA (1 μg/mL) in HEPES-Na buffer (100 mM, pH 7.4) containing CaCl_2_ (1 mM), MgCl_2_ (1 mM), DTT (100 μM) and Triton X-100 (150 μM) and incubated at 25°C for 18 h. Scale bar = 30 μm. (b) Fluorescence images of microdevice after loading the Suc-Ile-Glu-Pro-Asp-sHMRG (30 μM) and activated granzyme B (1 μg/mL) in HEPES-Na buffer (100 mM, pH 7.4) containing CaCl_2_ (1 mM), MgCl_2_ (1 mM), DTT (100 μM) and Triton X-100 (150 μM) and incubated at 25°C for 18 h. Scale bar = 30 μm. (c) Michaelis-Menten plot of Suc-Ile-Glu-Pro-Asp-sHMRG with activated granzyme B. The experiments were performed with 1 μg/mL enzyme in HEPES-Na buffer (100 mM, pH 7.4) containing CaCl_2_ (1 mM), MgCl_2_ (1 mM), DTT (100 μM) and CHAPS (0.1%) and incubating at 25°C for 60 min. The fluorescence increase rate was converted to the concentration change (M/s) using the fluorescence signal of sHMRG (1 μM) as a standard. Error bars represent S.D. (n = 4).

**[Comment]** The fluorogenic probes for PSA and granzyme B were designed and tested for their ability to detect single-molecule enzymatic activity using the conventional microdevice-based assay^12,14^. However, no fluorescent spots were observed for either enzyme. We considered that the major reason was the insufficient turnover numbers (*k*_cat_) of the probes, since single-molecule enzymatic assays typically require *k*_cat_ > 1 s⁻¹ to acquire the sufficiently detectable signals^10^. Optimization of probe structures or assay conditions may enable detection in conventional assays, but the ABP-based approach is more suitable for enzymes with lower turnover numbers or strict substrate recognition, for which the development of appropriate probes is challenging.


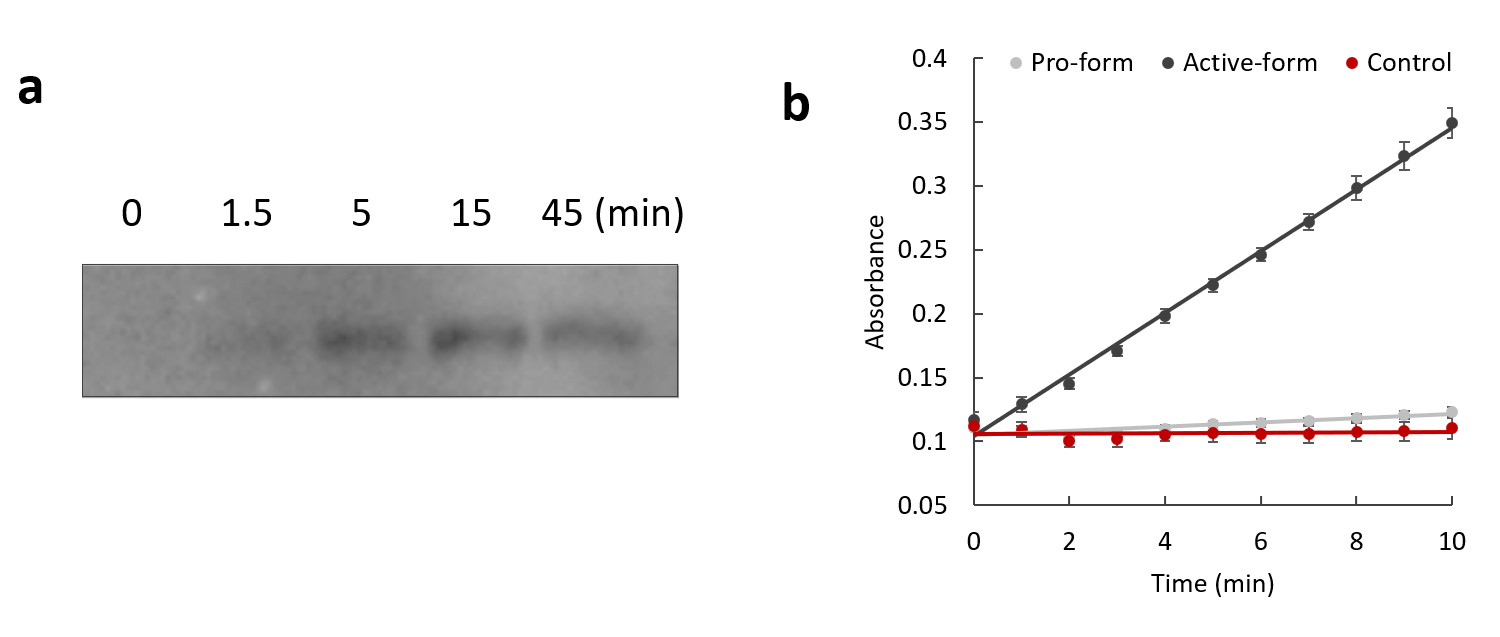


**Figure S2.** Optimization of PSA activation by thermolysin and confirmation of PSA activity using conventional absorption assay. Recombinant human pro-PSA (50 μg/mL) was mixed with recombinant thermolysin (1 μg/mL) in HEPES-Na buffer (100 mM, pH 7.4) containing NaCl (150 mM) and CHAPS (0.1%), and the mixture was incubated at 37°C for 1.5-45 min. 1,10-Phenanthroline was added with the final concentration of 20 mM. The activated enzyme solution was analyzed by Western blotting using Streptavidin-HRP-based chemiluminescence detection. (b) Absorbance (405 nm) change of Suc-RPY-*p*NA (4 mM) after mixing with activated PSA or pro-PSA (10 μg/mL) in HEPES-Na buffer (100 mM, pH 7.4) containing NaCl (150 mM) and CHAPS (0.1%) at 25°C. Error bars represent S. D. (n = 3).


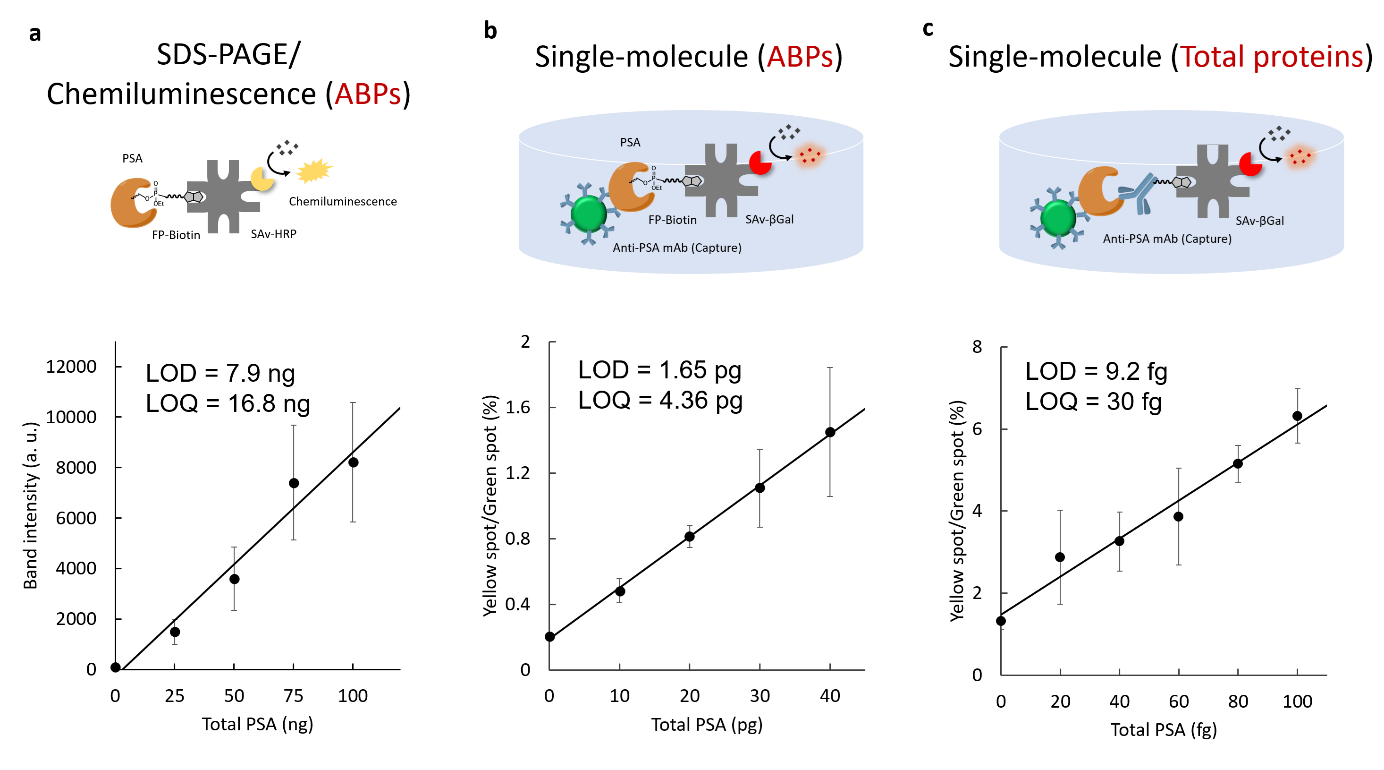


**Figure S3.** Comparison of limit of detection (LOD) and limit of quantification (LOQ) in detection of PSA in various systems. (a) The result of detection of activated PSA by ABP labeling and chemiluminescence-based detection by western blotting. The amount of PSA refers to pro-PSA used for activation. (b) The result of detection of activated PSA by ABP-based single-molecule assays. The amount of PSA refers to pro-PSA used for activation. (c) The result of detection of pro-PSA using digital ELISA platform^22^. Error bars represent S. D. (n = 3).

**[Comment]** To compare the detection sensitivity of conventional SDS-PAGE-based and single-molecule assays, we used the same protein samples labeled with FP-biotin and captured by a streptavidin-based system. Detection in the SDS-PAGE-based assay was performed using HRP chemiluminescence (**Figure S3a),** while detection in the single-molecule assay was performed using β-galactosidase in a microdevice (**Figure S3b**). For comparison between the digital ELISA platform for total PSA and the ABP-based assay for active PSA, the same source proteins were used, but the digital ELISA achieved a lower detection limit (**Figure S3c**). Since the capture unit (mAb beads) was identical in both assays, we considered that the difference arose not from the system itself but from the lower activation efficiency of pro-PSA by thermolysin, which is a non-specific protease and not the physiological activator of pro-PSA. Consequently, only a fraction of pro-PSA might have been converted to the active form that could be captured by FP-biotin and was detectable in ABP-based single-molecule assay (**Figure S3b**).


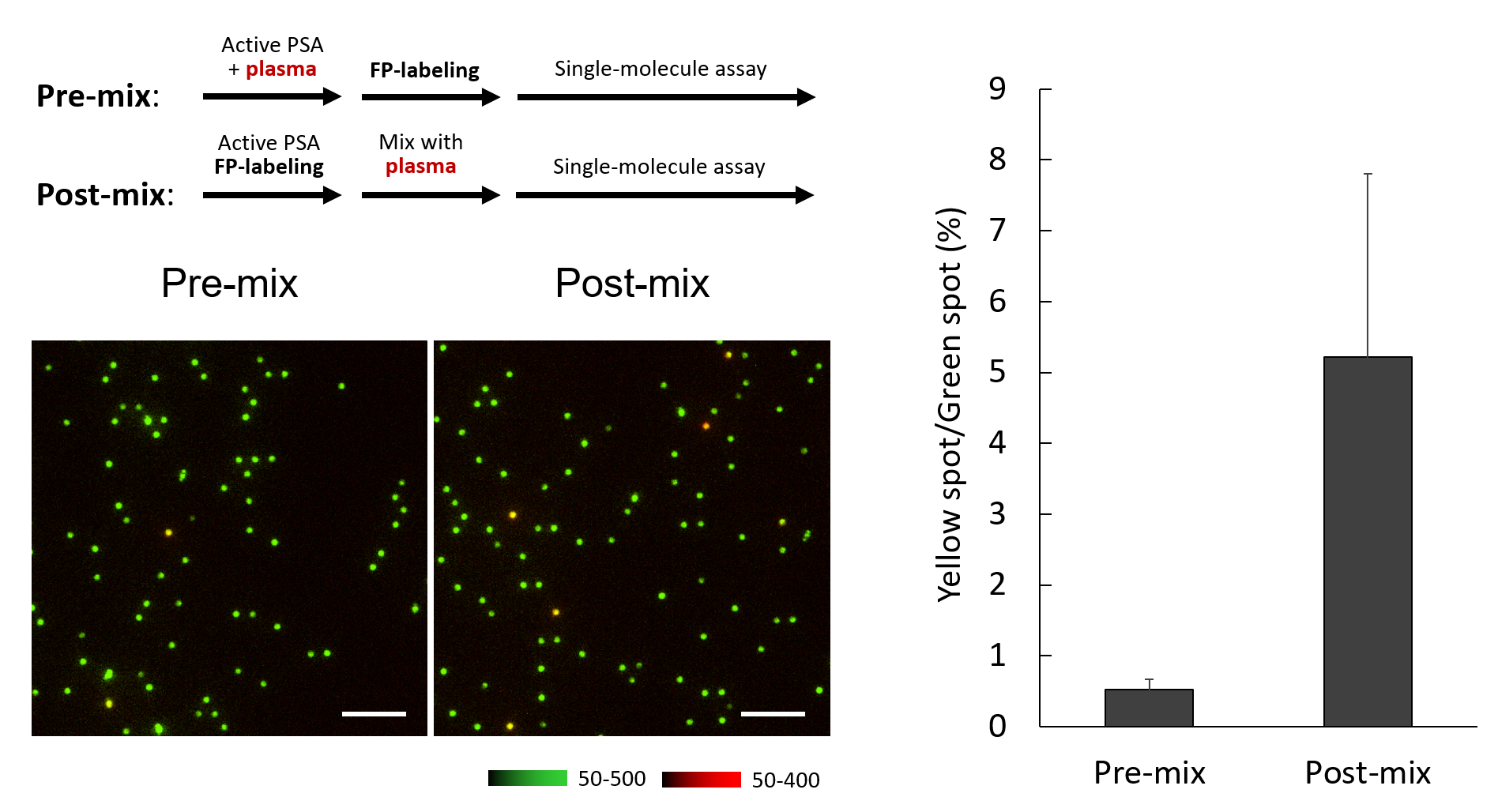


**Figure S4.** Inactivation of PSA activity labeling in plasma samples. For pre-mix conditions, 1/300-diluted plasma samples was mixed with 400 pg/mL activated PSA before labeling with FP-biotin. For post-mix conditions, 400 pg/mL activated PSA was labeled with FP-biotin and mixed with 1/300-diluted plasma samples. The detection method was same as that of **Figure 2b**. Error bars represent S. D. (n = 3). Scale bar = 30 μm.


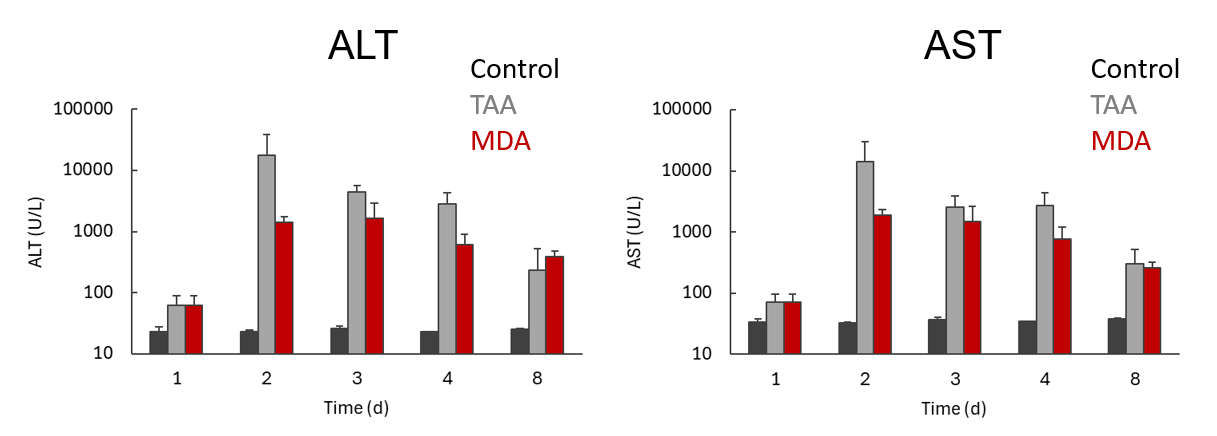


**Figure S5.** AST/ALT of liver damage samples. Error bars represent S. D. (n = 4 for control mice, n = 6 for TAA- and MDA-treated mice).


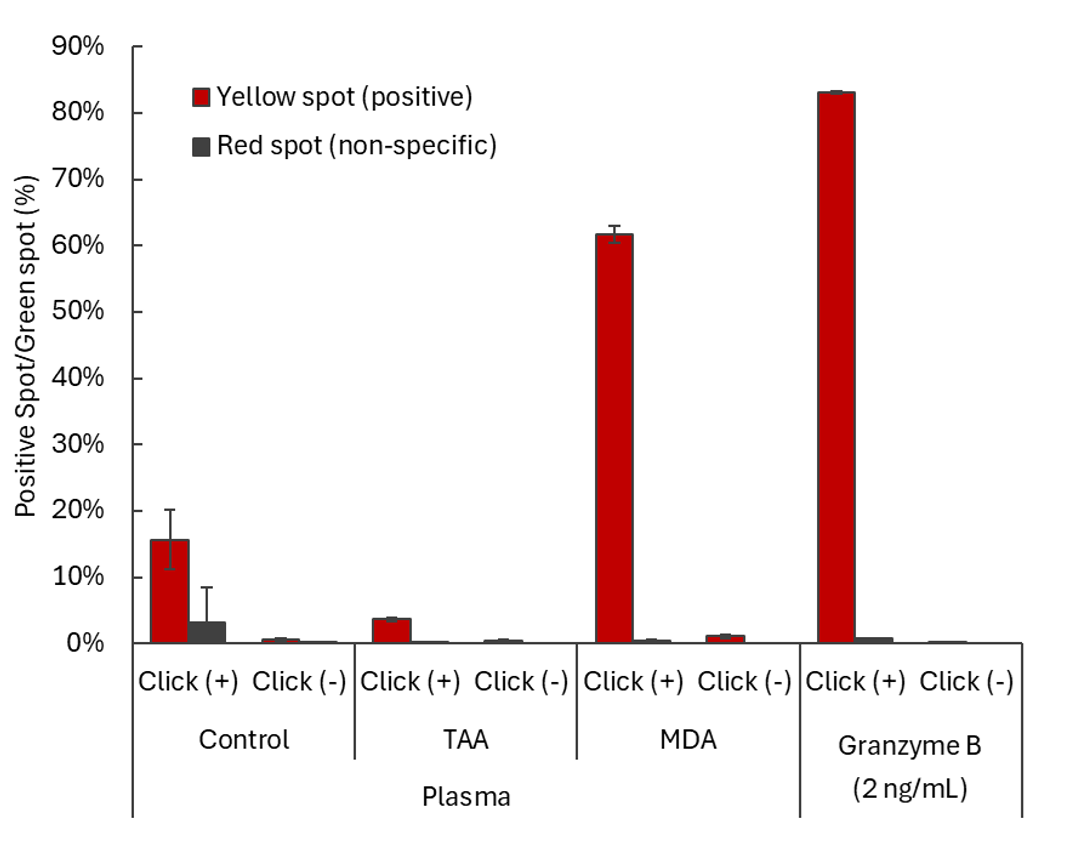


**Figure S6.** Conformation of click-dependent signal in blood samples. The experiment was same with that of **Figure 4d**. Click (-) is the condition in which the click-based elongation of the linker was performed without addition of CuSO_4_, BTTP, and sodium ascorbate. “Yellow spot” indicates the number of microchambers with green fluorescence (beads) and red fluorescence (β-galactosidase) were detected. “Red spot” indicates the number of microchambers in which only red fluorescence were detected, so it was generated as a non-specific binding of the conjugates. Error bars represent S. D. (n = 3).
